# The delay of psychiatric consultation in the moroccan framework

**DOI:** 10.1192/j.eurpsy.2023.1841

**Published:** 2023-07-19

**Authors:** H. Zarouf, M. Chtibi, S. Belbachir, A. Ouanass

**Affiliations:** Ar-razi University Psychiatric Hospital, Salé, Morocco

## Abstract

**Introduction:**

In 1830, Charles-Albert Perret-Porta, director of a Swiss mental asylum, said « it is especially at birth that alienation is curable ». This assertion dating back more than two centuries ago proves to be close to today’s clinical practice, as biological, social and psychological damage can be irreversible in the case of a delay in adequate psychiatric treatment. The emergence of the « Duration of Untreated Psychosis » concept is worth mentioning, as it is confirmed to be one of the determining factors in the psychotic disorders’ clinical outcome and prognosis.

Despite the risks incurred, many patients that suffer from psychiatric disorders still benefit from late adequate care, for various reasons.

**Objectives:**

The objective of this study is to identify the different causes of delay in psychiatric consultation in the Moroccan framework, in order to promote early intervention strategies by taking into account and acting on these different factors.

**Methods:**

This is a retrospective descriptive and analytic study carried out at the Arrazi University Psychiatric Hospital in Salé, having collected information from 101 patients (69,3% being inpatients).

The analytic part of the study was performed by JAMOVI.

**Results:**

The descriptive analysis showed that the mean age was 36 years ± 11,2. 73,3% were men. 68,3% of the patients were single, 18,8% were married, 11,9% were divorced and only 1 patient was a widow. 87,1% were living in the urban area. 23,8% attended higher education. 61,4% of the patients were unemployed. Patients were diagnosed with the following disorders, according to the DSM-5-R: Schizophrenia (73,3%), major depressive disorder (8,9%), schizoaffective disorder (6,9%), anxiety disorders (5,9%), bipolar disorder (4%), brief psychotic disorder (1%). The median of the first consultation period was 240 days [60,730]. The main causes of first consultation delay were: Lack of awareness about mental illness (34,7%), religious beliefs (33,3%), mental illness denial (10,7%).

There were no associations between the first consultation period and age (p=0.701), sex (p=0,929), diagnosis (Schizophrenia: p=0,420; anxiety disorders: p=0,569; Major depressive disorder: p=0,570; schizoaffective disorder: p=0,855; Bipolar disorder: p=0,624), human settlement (p=0,174).

**Conclusions:**

Mental health and psychiatry are still facing stigma in the Moroccan framework and many others developing countries, which hampers medical care for patients suffering mental illness, leading to both poorer prognosis and clinical outcomes.

Prevention campaigns promoting early intervention strategies should be a subject of concern among public health workers to overcome stigma in the perspective of improving medical care of mental illness.

**Disclosure of Interest:**

None Declared

